# The effect of dam construction on the movement of dwarf caimans, *Paleosuchus trigonatus* and *Paleosuchus palpebrosus*, in Brazilian Amazonia

**DOI:** 10.1371/journal.pone.0188508

**Published:** 2017-11-27

**Authors:** Zilca Campos, Guilherme Mourão, William E. Magnusson

**Affiliations:** 1 Embrapa Pantanal, Corumbá, Mato Grosso do Sul, Brazil; 2 Instituto Nacional de Pesquisa da Amazônia, Manaus, Amazonas, Brazil; Macquarie University, AUSTRALIA

## Abstract

Run-of-the-river hydroelectric dams cause changes in seasonal inundation of the floodplains, and this may cause displacement of semi-aquatic vertebrates present before dam construction. This study evaluated the movement of crocodilians before and after the filling of the Santo Antônio hydroelectric reservoir on the Madeira River in the Brazilian Amazon, which occurred in November 2011. We radio-tracked four adult male *Paleosuchus palpebrosus* and four adult male *Paleosuchus trigonatus* before and after the formation of the reservoir between 2011 and 2013. The home ranges of the *P*. *palpebrosus* varied from < 1 km^2^ to 91 km^2^ and the home ranges of the *P*. *trigonatus* varied from < 1km^2^ to 5 km^2^. The species responded differently to time since filling and water level in weekly movement and home range. However, overall the dam appears to have had little effect on the use of space by the individuals that were present before dam construction.

## Introduction

Historically, traditional hydroelectric dams with large reservoirs flooded extensive areas of terrestrial habitat and changed the conditions from lotic to lentic, which presumably led to the exclusion of many aquatic species present in the area before dam construction [1, 2]. During recent decades, there has been a tendency for the construction of run-of-the-river dams [3, 4], which inundate much smaller areas and maintain the lotic nature of most of the upstream sections.

Presumably, run-of-the-river dams have less drastic effects on the aquatic and semi-aquatic fauna than traditional dams. Nonetheless, even if species assemblages similar to those present before dam construction eventually reestablish, it is not known what happens to the individuals present when the dam fills and inundates local wetlands. Crocodilians are long lived and many species are territorial [5], so dam construction could lead to severe disruption of home ranges, possibly resulting in individuals abandoning the area and dispersing into areas where they experience increased risk of mortality, such as areas near dam turbines [6] or areas with less nesting habitat [7, 8].

Species of *Paleosuchus* are small crocodilians, the adults of which normally occupy relatively small home ranges [9, 10]. In some places near dam walls, the reservoir may completely destroy all suitable habitat previously occupied by the species [11]. However, most of the river system is not as strongly affected and it is possible that individuals could reestablish in areas close to their former home range

We used radio telemetry to study the movement of individuals *Paleosuchus palpebrosus* and *Paleosuchus trigonatus* before the filling of the Santo Antônio Hydroelectric Dam on the Madeira River in Brazilian Amazonia, and in equivalent annual periods one and two years after the filling of the dam. The study was concentrated around the Jaci River, which is a tributary of the Madeira River in the central part of the area inundated by the reservoir. This allowed us to evaluate the effects of water level in the three periods and to determine to what extent dam filling resulted in changes in short-term rates of movement and displacement of home ranges.

## Materials and methods

### Ethics statement

The research project was approved by the Brazilian Environmental Agency (IBAMA permit N°. 017/02) and by the Chico Mendes Institute for Biodiversity Conservation (ICMBio permanent license N°. 13048–1) for capture and marking caimans (Normative regulation N°. 154/2007). All procedures followed ethical practices for animals approved by Committee on the Ethics of Animal Research of the Brazilian Agricultural Research Organization (Embrapa N° 009/2016). No biological material, such as blood or tissue, was collected in this study. These species are classified by the International Union for Conservation of Nature (IUCN) as Lower Risk, least concern for conservation.

### Study site and sampling design

The study was undertaken in the area affected by the Santo Antônio hydroelectric dam (SAHD) on the Madeira River in western Amazonia. The general area and dam characteristics have been described in a previous study [11]. The climate in the region is hot and humid with rainy season from October to April [12]. The water level of the Jaci River before filling ranged from 0.90 m to 8.00 m throughout the year, and after filling ranged from 9.33 to 12.69 m.

The two species of *Paleosuchus* are small crocodilians that occupy small streams in continuous forest and inundated forests near large rivers in Amazonia [13], and many of these environments close to large rivers are permanently flooded by hydroelectric dams [11]. The two species engage in terrestrial movements in riparian areas and spend extended periods in terrestrial retreats, mainly in dry and cool periods [8, 14, 15].

Between January and August 2011, we captured dwarf caimans from a boat using a steel-cable noose attached to a pole and attached radio transmitters (MOD 400, Telonics) to the tails of four adult male *P*. *trigonatus* and four adult male *P*. *palpebrosus*. The movements of these individuals were monitored (8°47'00"S, 63°56'00"W) before and after the formation of the reservoir of the Santo Antônio hydroelectric dam. Snout-vent length (SVL cm) of the *P*. *trigonatus* ranged from 70.0 to 87.0 cm and body mass from 9.0 to 15.0 kg. For *P*. *palpebrosus*, SVL ranged from 69.0 to 82.5 cm and body mass from 9.0 to 18.0 kg (Table 1).

**Table 1:** Snout-vent length (SVL cm) and body mass of *Paleosuchus palpebrosus* (Pp) and *Paleosuchus trigonatus* (Pt) used in the study. The code for the individual is the same as that used on the figures.

Code	Date	Species	SVL (cm)	Body mass (kg)
8	01/02/2011	Pp	79.5	12.0
09	01/02/2011	Pp	80.0	13.0
10	22/06/2011	Pp	69.0	9.0
66	27/072011	Pp	82.5	18.0
111	31/07/2011	Pt	77.2	11.0
33	02/08/2011	Pt	70.0	9.0
44	22/06/2011	Pt	72.0	8.0
55	24/06/2011	Pt	87.0	15.0

Surveys to locate animals with transmitters were undertaken at weekly intervals throughout the study period by boat and/or on foot. However, to maintain comparability, and take into account possible seasonal variation in space use, we analyzed only data for the calendar months sampled pre-inundation for each animal for both the pre- and post-inundation periods. Although this required discarding data in post-inundation periods, it did not affect the general location or size of home ranges. Data for all surveys are available from the Dryad Digital Repository titled “Data from: Movement of the *Paleosuchus palpebrosus* and *Paleosuchus trigonatus*” DOI:doi:10.5061/dryad.1sj40 and Laboratory Protocols ≤ https://www.protocols.io/view/the-movement-of-the-paleosuchus-palpebrosus-and-pa-jzkcp4w.

We divided the sampling period into three phases. Phase 1 was the period of monitoring before dam filling (Feb-Nov/2011); phases 2 and 3 included the same monitoring months in the following years (Phase 2 = Feb-Nov/2012; Phase 3 = Feb-Nov/2013).

### Data analysis

All analyses were done in the R statistical language environment, version 2.1.11 [16]. We examined graphically the evolution of the distance from the origin, defined as distance from the capture location of the caiman, through time. We used linear mixed-effects models (lme) adjusted by maximum likelihood (ML) to test whether caiman movement, indexed by the distance moved between consecutive locations, differed among phases. Candidate model predictors were water level (fixed factor), phase nested within individuals (fixed), species (fixed) and individuals (random). We adjust three models with different degrees of complexity (“S1 and S2 Tables”) using the lme function of the nlme package [17]. The first model included as explanatory variables only the overall mean and the nested structure (phase within individuals). The second model included the phase, the interaction between phase and caiman species and the nested structure. The full model included phase, the interaction between phase, caiman species and water level, and the nested structure. We assessed the significance of our models by applying an ANOVA and selected the best model using the Akaike Information Criterion (AIC). Once the best model was selected, we re-ran it using restricted maximum likelihood (REML) to obtain the parameter estimates [18].

Home ranges were estimated for each caiman in each phase by the fixed-kernell 95% technique [19] with the package adehabitatHR [20]. We used the Friedman non-parametric analysis of variance to test for differences in the size of home ranges between phases for each species.

The map of the reservoir was produced in the Argis Desktop 10 program (www.esri.com/argis) by Santo Antônio Energia (SAE). The Spring 5.5.0 program (www.dpi.inpe/spring) was used by Z. Campos and collaborators from Embrapa to plot the locations of caimans. Permission to publish the maps was granted by SAE and Embrapa.

## Results

**Fig 1:**
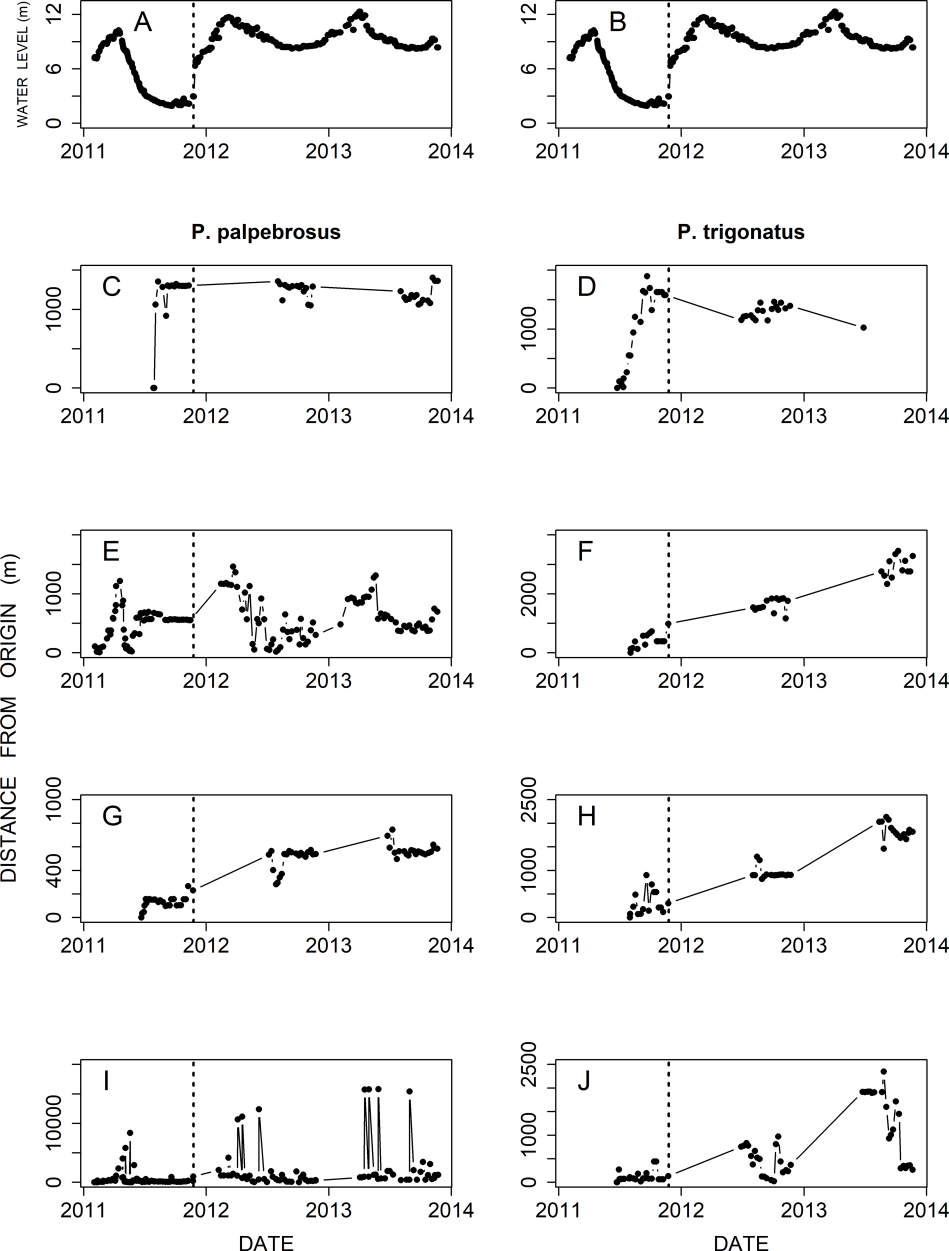
Water-level variation of the Jaci River, tributary of the Madeira River, in the reservoir area of the Santo Antônio Dam (upper graphs). Distances moved from the point of origin by four *Paleosuchus palpebrosus* and four *Paleosuchus trigonatus* at different times during the study. The dam was filled in November 2011.

Displacement from the origin through the phases did not appear to differ between caiman species (Fig 1). Some individuals displaced little from the capture local (origin) (Fig 1C and 1D), some moved continually away from the origin between the phases (Fig 1F, 1G and 1H). Others returned repeatedly to the original point of capture (Fig 1I and 1J). However, one *P*. *palpebrosus* (Fig 1I) was located one month after the end of the study period about twenty kilometers from the original location.

**Fig 2:**
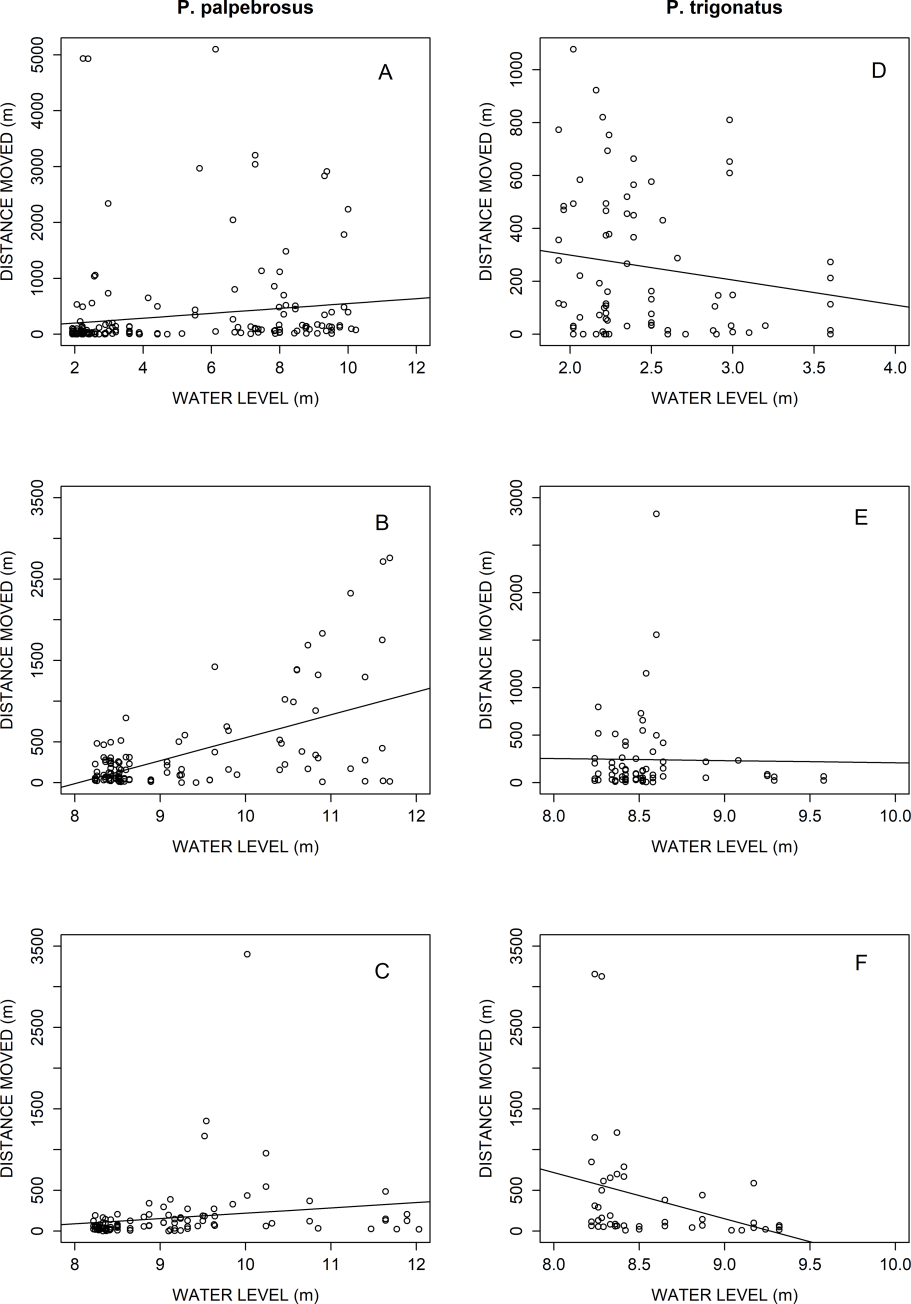
Distances moved between relocations (m) by phases in relation to water level for four male *Paleosuchus palpebrosus* (A, B, C) and four male *Paleosuchus trigonatus* (D, E, F).

ANOVA indicated that the full model explained more variability in the response variable than the other competing models, and the full model AIC value was 30 AIC units lower than the previous competing models (Table 2). The best model to explain the distance moved by the caimans indicated that the responses of the caimans to the phases were complex and largely depended of the species (Table 3). For both species, effects of phase 2 differed from the other phases (p < 0.001) and the *P*. *trigonatus* moved more than the *P*. *palpebrosus* during phase 3 (p = 0.022, “S1 Fig”). The caiman species responded differently to the interaction of phases and water level. *P*. *palpebrosus* (Fig 2A, 2B and 2C) tended to increase their distance moved between locations with the increase in water level during phase 1 (p = 0.008) and phase 2 (p > 0.001) and ceased to do so in phase 3 (p = 0.252). *P*. *trigonatus* (Fig 2D, 2E and 2F) did not respond to the increase in the water level during phases 1 and 2 (p = 0.530 and p = 0.923, respectively), but moved less between relocations in phase 3 (p = 0.017).

**Table 2:** ANOVA table comparing the three competing models for caiman movement.

Model	df	AIC	BIC	logLik	Test	L.Ratio	P
1	4	9060.023	9077.468	-4526.011			
2	9	9064.424	9103.676	-4523.212	1 vs 2	5.59814	0.3473
3	15	9034.056	9099.475	-4502.028	2 vs 3	42.36862	<0.0001

**Table 3:** Results of the linear mixed-effects model to explain the movements between locations of four male *P*. *palpebrosus* (sppp) and four male *P*. *trigonatus* (sppt) radio-monitored in the Santo Antônio Hydroelectric Dam (SAHD), Brazil.

Predictors	Estimate	SE	DF	t	P
(Intercept)	113.275	107.5850	550	1.0529	0.293
phase2	-2376.992	483.1832	10	-4.9194	<0.001
phase3	-546.593	530.5381	10	-1.0303	0.327
phase1:sppt	374.751	393.9953	10	0.9512	0.364
phase2:sppt	2701.294	2086.0184	10	1.2950	0.224
phase3:sppt	5682.221	2099.3907	10	2.7066	0.022
phase1:sppp:wl	43.661	16.3773	550	2.6659	0.008
phase2:sppp:wl	281.569	51.2162	550	5.4977	<0.001
phase3:sppp:wl	65.183	56.8904	550	1.1458	0.252
phase1:sppt:wl	-94.326	150.1829	550	-0.6281	0.530
phase2:sppt:wl	-22.854	237.2610	550	-0.0963	0.923
phase3:sppt:wl	-566.343	236.7461	550	-2.3922	0.017

Model predictors were phase (1 = pre-filling period, 2 = first year post-filling period and 3 = second year post-filling of the SAHD), the interaction between phase, caiman species and water level, and the phase nested within individuals (random). SE = standard error, DF = degrees of freedom.

**Fig 3:**
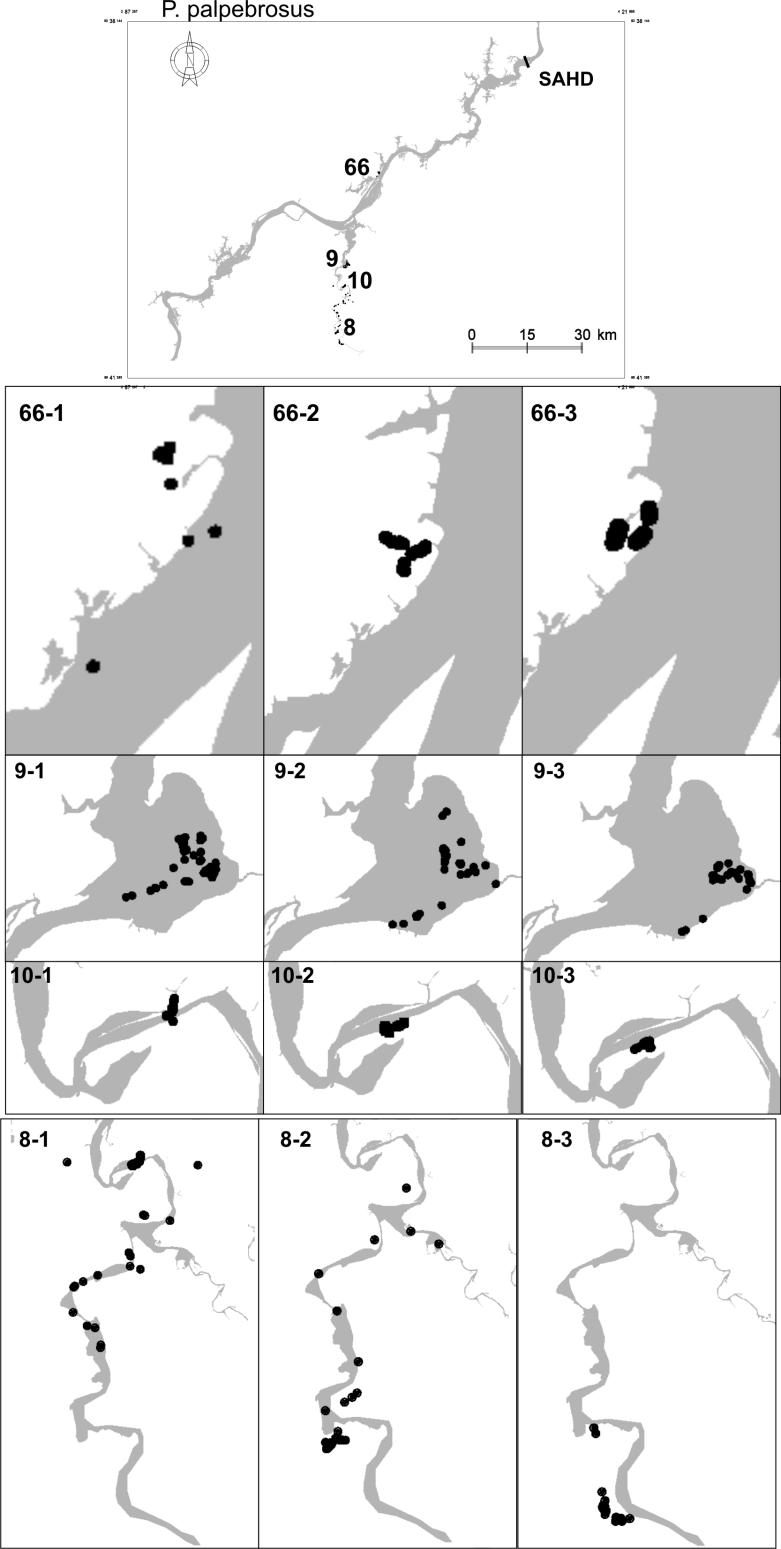
Map locations of four individuals of *Paleosuchus palpebrosus* that were first captured in the locations shown in the upper map. Each horizontal panel represents one individual in different periods related to dam filling. The identifying numbers on each map indicate the individual (before the stroke) and the year of the observations (after the stroke). The map of the reservoir was produced in the Argis Desktop 10 program (www.esri.com/argis) by Santo Antônio Energia (SAE). The figure was created in the Spring 5.5.0 program (www.dpi.inpe/spring).

**Fig 4:**
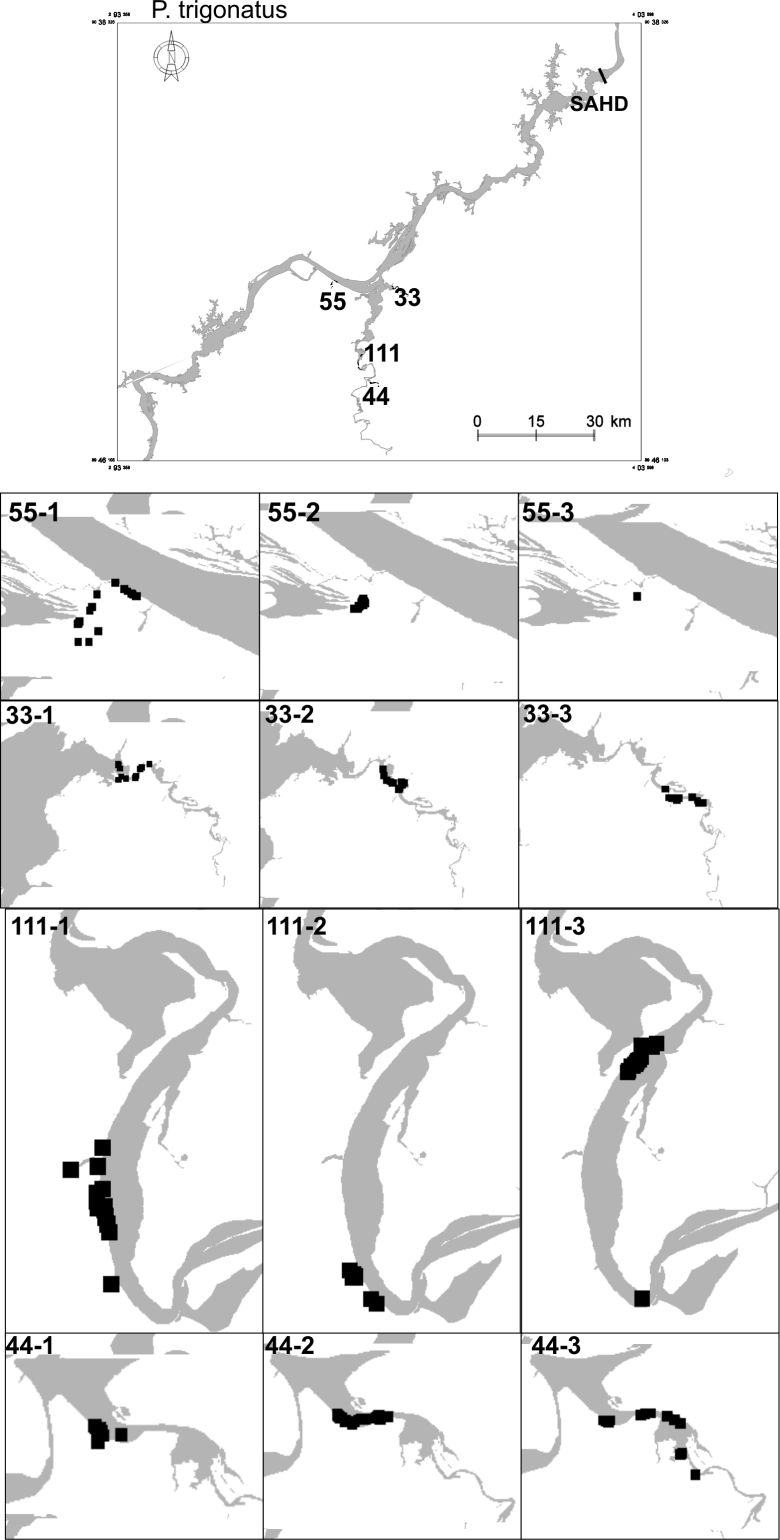
Map locations of four individuals of *Paleosuchus trigonatus* that were first captured in the locations shown in the upper map. Each horizontal panel represents one individual in different periods in relation to dam filling. The identifying numbers on each map indicate the individual (before the stroke) and the year of the observations (after the stroke). The map of the reservoir was produced in the Argis Desktop 10 program (www.esri.com/argis) by Santo Antônio Energia (SAE). The figure was created in the Spring 5.5.0 program (www.dpi.inpe/spring).

Home ranges of *P*. *palpebrosus* (Fig 3) ranged from < 1 km^2^ to 90.94 km^2^ (median = 1.22 km^2^, IQR = 2.88) and differed between phases (χ^2^ = 6, df = 2, p = 0.049). The home ranges of the *P*. *trigonatus* (Fig 4) ranged from < 1 km^2^ to 4.89 km^2^ (median = 0.98 km^2^, IQR = 1.99) and did not differ significantly between phases (χ^2^ = 6, df = 2, p = 0.472).

## Discussion

Movement of crocodilians is influenced by water level and seasonal variation in aquatic habitats [21, 22]. Hydroelectric dams modify the water-level regime [23] but there have been no studies on how caimans respond to such changes.

In this study, mean rates of movement differed among years for *P*. *trigonatus*, and among years, among individuals, and depending on water level for *P*. *palpebrosus*. However, all factors affected home ranges for both species, indicating that individuals are affected by, and respond idiosyncratically to, dam filling. The margins of major Amazonian rivers are subject to deep and irregular yearly flooding, so flooded-forest organisms have to be tolerant of the sorts of changes caused by run-of-the-river dams. The higher mean water levels could eliminate important resources locally, and we had expected some of the caimans to be displaced long distances, but, in general, this did not happen. The home range of one *P*. *palpebrosus* covered 91 km^2^ before the dam was constructed, and the mean home-range size of the *P*. *trigonatus* in this study before dam construction was 4.89 km^2^, which is similar to that found for the species in an area of rainforest in central Amazonia [7].

Despite the fact that dam filling resulted in displacement of most of the individuals of both species from their original home ranges, all individuals reestablished close to where they were originally captured and none engaged in long-range movements that might result in risk of mortality due to the dam turbines. At least in the short term, the dam appears to have had little effect on the use of space by the individuals that were present before dam construction. This is good news, both from the point of view of conservation and animal welfare. However, long-term studies are necessary to determine whether the caimans will be able to survive and breed in their new home ranges.

## Supporting information

S1 Fig Contrast plot of the best model (see text) representing the moved distance between relocations (m) by caiman species. Pp = *P*. *palpebrosus*; pt = *P*. *trigonatus*. The plot also shows the 95% confidence band (grey) and the prediction linee (blue).
(TIF)

S1 Table The three competing linear mixed-effects models to exam whether the different phases explained the differences in caiman movement, indexed by the distance moved between consecutive locations. dist = distance moved between locations; id = id of each monitored caiman; wl = water level, and sp = species of monitored caiman. (DOCX)

S2 Table ANOVA table comparing the three competing models for caiman movement. (DOCX)
