# Supplementary figures and images for: The effect of dam construction on the movement of dwarf caimans, Paleosuchus trigonatus and Paleosuchus palpebrosus, in Brazilian Amazonia
Source: PLoS One. 2017 Nov 27;12(11):e0188508. doi: 10.1371/journal.pone.0188508 (PMC5703545; doi:10.1371/journal.pone.0188508)

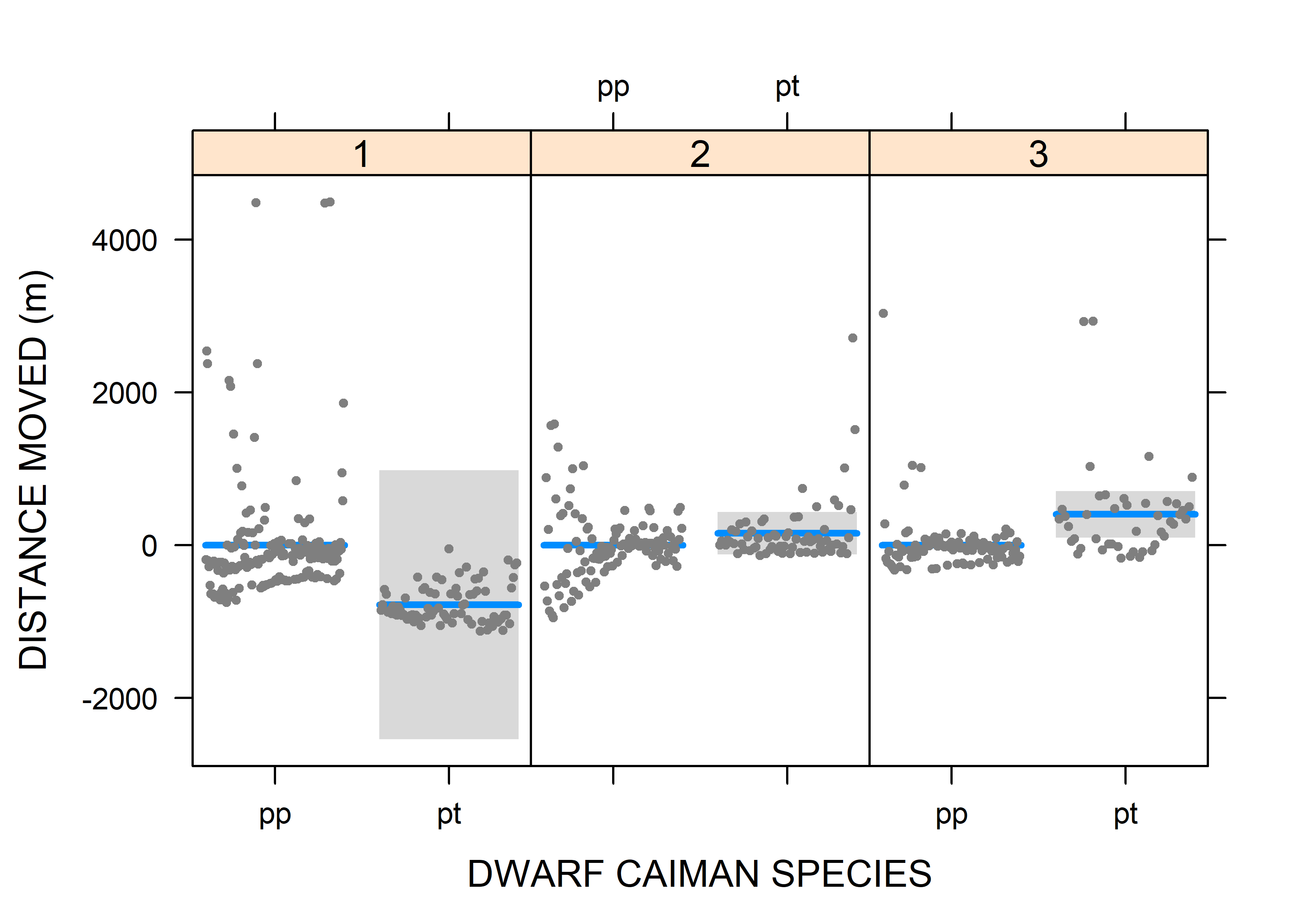

Supplement: S1 Fig — Pp = P. palpebrosus; pt = P. trigonatus. The plot also shows the 95% confidence band (grey) and the prediction linee (blue). (TIF) [file pone.0188508.s001.tif]
