# Supplementary material for: The effect of dam construction on the movement of dwarf caimans, Paleosuchus trigonatus and Paleosuchus palpebrosus, in Brazilian Amazonia
Source: PLoS One. 2017 Nov 27;12(11):e0188508. doi: 10.1371/journal.pone.0188508 (PMC5703545; doi:10.1371/journal.pone.0188508)
Supplement: S1 Table — (DOCX) [file pone.0188508.s002.docx]

S1 Table. The three competing linear mixed-effects models to exam whether the different phases explained the differences in caiman movement, indexed by the distance moved between consecutive locations. dist = distance moved between locations; id = id of each monitored caiman; wl = water level, and sp= species of monitored caiman.

| Model structure | AIC |
| --- | --- |
| model1 <- lme(dist~1,random=~1\|id/phase,data=two.spp,method='ML') | 9060.023 |
| model2 <- lme(dist~ phase + phase:sp,random=~1\|id/phase,data=two.spp,method='ML') | 9064.424 |
| **model3 <- lme(dist~phase+ phase:sp+ phase:sp:wl,random=~1\|id/phase,data=two.spp,method='ML')** | **9034.056** |
