# Supplementary material for: The effect of dam construction on the movement of dwarf caimans, Paleosuchus trigonatus and Paleosuchus palpebrosus, in Brazilian Amazonia
Source: PLoS One. 2017 Nov 27;12(11):e0188508. doi: 10.1371/journal.pone.0188508 (PMC5703545; doi:10.1371/journal.pone.0188508)
Supplement: S2 Table — (DOCX) [file pone.0188508.s003.docx]

S2 Table. ANOVA table comparing the three competing models for caiman movement.

| Model | df | AIC | BIC | logLik | Test | L.Ratio | p |
| --- | --- | --- | --- | --- | --- | --- | --- |
| 1 | 4 | 9060.023 | 9077.468 | -4526.011 |  |  |  |
| 2 | 9 | 9064.424 | 9103.676 | -4523.212 | 1 vs 2 | 5.59814 | 0.3473 |
| 3 | 15 | 9034.056 | 9099.475 | -4502.028 | 2 vs 3 | 42.36862 | <.0001 |
